# Supplementary figures and images for: Plasma proteome signature of canine acute haemorrhagic diarrhoea syndrome (AHDS)
Source: PLoS One. 2024 Feb 8;19(2):e0297924. doi: 10.1371/journal.pone.0297924 (PMC10852219; doi:10.1371/journal.pone.0297924)

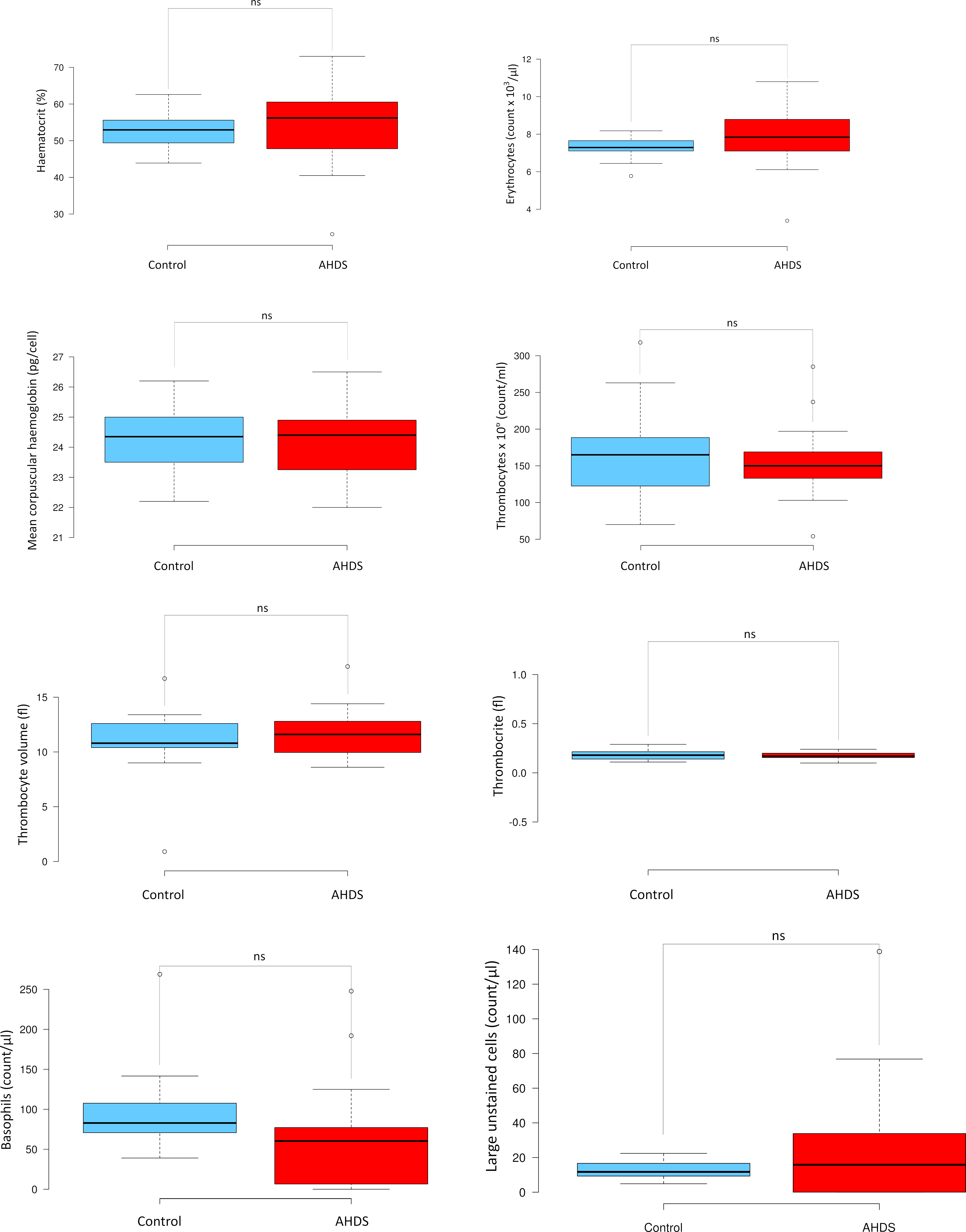

Supplement: S1 Fig — The boxplots illustrate notable variations in blood cell markers between these two groups. Statistical distinctions were assessed using the student’s t-test for samples with equal variance or the Welch test for samples with differing variances. Statistical significance was attributed to values with p < 0.05. (TIF) [file pone.0297924.s001.tif]

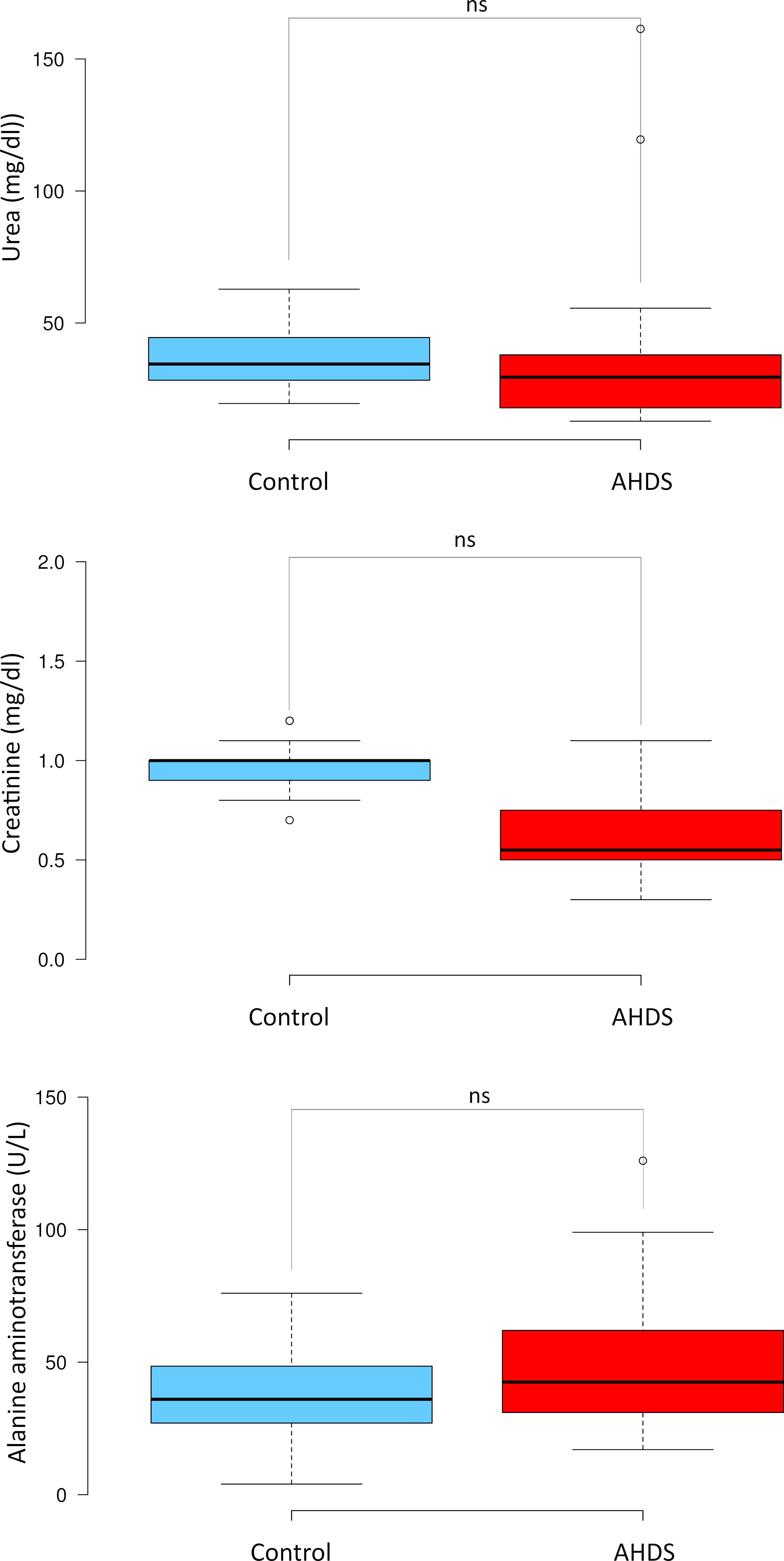

Supplement: S2 Fig — The boxplots highlight noteworthy variations in specific plasma biochemistry markers between the AHDS and control groups. Statistical differences were evaluated using either the student’s t-test for samples with equal variance or the Welch test for samples exhibiting varying variances. Statistical significance was attributed to values with p < 0.05. (TIF) [file pone.0297924.s002.tif]
